# Treatment of Ozœna

**Published:** 1875-12

**Authors:** 


Treatment of Ozæna by Injections of Chloral.—M. Crequy reports the cure of an obstinate case of ozæna by the injection of a solution of chloral. He simply made a siphon of an india-rubber tube by putting one end to the injection and the other well up into the nasal cavity. The disease had resisted injections of tannin, phenon, corrosive sublimate, etc., but was cured in a short time by this method.—*Bull. Gen. de Therap.*

